# Myeloid-Derived Suppressor Cells: The Expanding World of Helminth Modulation of the Immune System

**DOI:** 10.3389/fimmu.2022.874308

**Published:** 2022-06-10

**Authors:** Mary M. Stevenson, Rajesh M. Valanparambil, Mifong Tam

**Affiliations:** ^1^ Department of Microbiology and Immunology, McGill University, Montreal, QC, Canada; ^2^ Division of Experimental Medicine, Department of Medicine, McGill University, Montreal, QC, Canada

**Keywords:** myeloid-derived suppressor cells, MDSC, helminths, gastrointestinal nematodes, immune suppression, immunophenotyping

## Abstract

Infection with helminths or parasitic worms are highly prevalent worldwide especially in developing regions. Helminths cause chronic infections that are associated with suppression of immune responses to unrelated pathogens, vaccines, and by-stander antigens responsible for dysregulated immune responses as occurs in diseases such as allergies. Helminths use multiple mechanisms to modulate the immune system to evade the highly polarized type 2 immune response required to expel adult worms and for immunity to reinfection. Anthelmintic drugs are efficient in reducing adult worm burdens in helminth-infected individuals, but resistance to these drugs is rapidly increasing and vaccines against these pathogens are not available. Emerging evidence indicate that helminths induce myeloid-derived suppressor cells (MDSC), originally described in tumor-bearing mice and cancer patients. MDSC are a heterogenous population of immature cells that consist of two distinct sub-populations, polymorphonuclear (PMN)-MDSC and monocytic (M)-MDSC based on morphology and phenotype. MDSC suppress the function of T cells and other innate and adaptive immune cells including NK cells and B cells. During cancer or infection with bacteria or viruses, there is marked expansion of MDSC. Furthermore, the frequencies of MDSC correlate inversely with the prognosis and survival of tumor-bearing hosts as well as bacterial and viral burdens, persistence, and outcome in infected hosts. Currently, there is a paucity of data on MDSC and helminth infections. Here, we provide a survey of the evidence accumulated so far that overall support a role for MDSC in modulating immune responses during helminth infections. We review data from studies in various helminths, including those that infect humans. Finally, we summarize the progress to date in understanding the role of MDSC in helminth infections and briefly discuss potential host-directed strategies to target MDSC-mediated suppression of immune responses to helminths in favor of development of immunity to eliminate adult worms and possibly induce protection against reinfection.

## Introduction

Myeloid-derived suppressor cells (MDSC), a heterogenous population of immature cells with potent immunosuppressive properties, were first described more than 30 years ago (1). MDSC were initially described in cancer based on their ability to inhibit anti-tumor T cell responses. Recently, these cells have been implicated in regulating immune responses in other diseases, including chronic inflammation, infection, sepsis, autoimmunity, and graft versus host disease. MDSC also appear to play important roles in normal physiological processes including pregnancy and possibly in newborns (2). Evidence of the clinical significance of MDSC in cancer and other diseases is rapidly increasing resulting in the development of host-directed strategies to mitigate or enhance their effects on immune responses and in pathological and physiological conditions, respectively.

Infections with helminths or parasitic worms are highly prevalent worldwide and cause infections in the poorest regions of the world primarily in tropical and sub-tropical countries. Helminth infections, including infections with soil-transmitted helminths (STHs), are among the most important neglected tropical diseases (NTDs) (3). More than 1.5 billion people or about 24% of the world’s population are infected with STHs including the hookworm species *Necator americanus* and *Ancylostoma duodenale* ^1^. Helminths are classified based on the shape of the adult worm as trematodes (e.g. flukes), cestodes (e.g. tapeworms), and nematodes (e.g. round worms). These parasites typically establish chronic infections and can continue to reproduce in their hosts, in some cases up to 20 years, unless treatment with an anthelmintic drug is administered (4). Helminth species also infect livestock including cattle, sheep, and pigs with significant impacts on the economy as well as food security. Importantly, helminths have evolved multiple mechanisms to evade the host type 2 immune response required for eliminating adult worms (4). These mechanisms include the ability of helminths to induce an immunoregulatory network involving several cell populations and cytokines with immunomodulatory effects or *via* their ability to produce excretory-secretory (ES) products that modulate immune cells. Indeed, ES products from various helminths have been identified and their modulatory effects demonstrated ex vivo on immune cells from both mice and humans and their therapeutic potential assessed *in vivo* in animal models of immune-mediated diseases such as allergy, inflammatory bowel disease, and multiple sclerosis (5). MDSC may represent an additional mechanism used by helminths to modulate host immune responses.

Here, we provide a survey of the evidence accumulated so far that support a role for MDSC in modulating immune responses during helminth infections. We review data from studies in various helminths, including those that infect humans. Finally, we summarize what is known to date about the role of MDSC in helminth infections and briefly discuss potential strategies to target MDSC-mediated suppression of immune responses to helminths in favor of development of immunity to eliminate adult worms and possibly induce protection against reinfection.

### Biology, Pathogenesis, and Immunity to Helminths

Helminth parasites are diverse and have unique, often complex, life cycles that influence the pathogenesis and diseases they cause and the immune responses they induce. In general, these parasites are transmitted *via* skin contact with contaminated soil or water or *via* the oral-fecal route. To better appreciate the impact of pathogenic helminths and their biology, readers are encouraged to consult the information on global health and NTDs on the Centers for Disease Control (CDC) website ^1^ which provides a wealth of information about helminths, their life cycles, the diseases they cause, and treatment.

Unlike infections with parasites such as *Plasmodium falciparum*, a causative agent of malaria responsible for millions of deaths worldwide, helminth infections including infections with STHs do not kill their hosts ^1^. However, helminth infections often cause severe morbidity. Morbidity associated with helminth infections is directly related to the adult worm burden in the colonized tissues of infected individuals and the chronicity of the infection. For example, light parasite burdens after infection with gastrointestinal (GI) nematodes are usually asymptomatic, while heavy parasite burdens are associated with symptoms ranging from general malaise, weakness, and intestinal distress (diarrhea and abdominal pain) as well as malnutrition and anemia. Chronic schistosomiasis is associated with enlargement of the liver and spleen, while severe filarial disease can lead to disfiguring enlargement of lymphatic tissues. Helminth infections have enormous impact on the health of children in developing countries. Infected children are especially prone to nutritional and physical impairments with adverse effects on growth and cognitive development (3). Anthelmintic drugs can reduce worm burdens and decrease morbidity, but immunity to re-infection does not develop after elimination of adult worms and drug resistant parasites are becoming of increasing concern (3). Unfortunately, there are no effective vaccines against parasitic helminths. Vaccine development is difficult because there is not a clear understanding of how the host immune system intersects with the complex life cycles of helminths.

Helminths induce a highly polarized Th2 immune response characterized by secretion of IL-4, IL-5, IL-9, and IL-13 (6–8). These cytokines mediate the expansion and activation of numerous cell types including alternatively activated macrophages, eosinophils, basophils, mast cells, and type 2 innate lymphoid cells as well as production of high levels of polyclonal IgE and parasite-specific IgE and IgG1. Altogether, these responses contribute to control of primary infection and prevention of reinfection. Moreover, these responses ensure parasite burdens are held in check to promote both host and pathogen survival. As mentioned above, helminth parasites suppress immune responses to unrelated pathogens and disease-promoting immune responses associated with allergy and autoimmunity (4). Remarkably, the strong Th2 responses induced by helminths are accompanied by potent immunoregulatory pathways including tolerogenic dendritic cells, alternatively activated macrophages, regulatory B cells, and regulatory T cells, and the cytokines IL-10 and TGF-β (4, 9). What’s more, helminth antigens and secreted molecules exert modulatory effects on immune cells particularly antigen presenting cells. For example, GI nematodes produce excretory-secretory (ES) products that modulate the antigen presenting function of dendritic cells (DC) and the function of bone marrow-derived macrophages (10, 11). Down-regulation of IL-12 secretion and co-stimulatory molecule expression and modulation of DC function by ES from the GI nematode *Heligmosomoides polygyrus bakeri* result in inhibition of antigen-specific CD4^+^ T cell proliferation and dampen Th1 as well as Th2 responses to by-stander antigen (10). Furthermore, ES-treated DC can induce the differentiation of CD4^+^CD25^+^Foxp3^-^ regulatory T cells that secrete high levels of IL-10 and suppress effector CD4^+^ T cell proliferation and IFN-γ secretion *via* an IL-10-dependent mechanism. As will be described below, helminths are also highly adept at inducing the expansion of MDSC.

### MDSC: Origin, Phenotype, and Function

Together with mononuclear phagocytes, DC, and polymorphonuclear cells (PMN) including neutrophils, eosinophils, basophils, and mast cells, MDSC are derived from myeloid progenitors (1). MDSC are heterogenous cells, which are considered to be immature, express CD11b and Gr1. MDSC consist of two sub-populations: granulocytic or PMN MDSC (PMN-MDSC) and monocytic MDSC (M-MDSC). The lack of distinctive phenotypic and morphological properties of MDSC has been a major challenge for researchers studying the roles of MDSC in humans as well as in mice. PMN-MDSC are currently identified in mice as CD11b^+^Ly6G^+^Ly6C^lo^ while M-MDSC are CD11b^+^Ly6G^-^Ly6C^hi^ cells (2, 12). It should be noted that conventional PMN and mononuclear phagocytes also express similar markers. Thus, expression of Ly6C and Ly6G is not limited to MDSC.

Distinguishing between neutrophils and PMN-MDSC is difficult in humans, since these cell populations share similar cell surface markers, including CD11b, CD15, and CD66b (2). However, human PMN-MDSC can be purified using a low density gradient while purification of PMN requires a high density gradient. Importantly, lectin-type oxidized LDL receptor 1 (LOX1) has been identified as a specific marker expressed by human PMN-MDSC in cancer patients (13). HLA-DR is expressed by human monocytes in contrast to human M-MDSC, that are HLA-DR^lo^/^-^ (2, 14). CD84, a member of the signaling lymphocyte activation molecule (SLAM) family, is expressed by hematopoietic cells and modulates immune responses. CD84 has been shown to be expressed by both PMN-MDSC and M-MDSC in patients with various cancers and in tumor-bearing mice (15, 16).

Importantly, single cell transcriptomics has allowed high resolution profiling of cells types including MDSC in tumors in mice and humans. However, studies using this approach are relatively limited likely due to the problem of isolating MDSC from tumors (17). Studies by Halaby and colleagues profiled FACS-sorted CD11b^+^Gr1^+^MHCII^low-neg^ MDSC from B16F10 tumors in mice and showed that *Il1b* is highly expressed in this cell population (18). A second study that profiled gene expression in MDSC in the spleen of mice with breast cancer confirmed *Il1b* as well as *Arg2* are highly up-regulated in PMN-MDSC (15). The later study also identified CD84 as a specific cell surface marker for MDSC in tumor-bearing hosts. Transcriptional profiling of tumors and normal tissues from patients with non-small cell lung cancer or pancreatic ductal adenocarcinoma revealed multiple overlap in highly expressed genes in MDSC and PMN and monocytes and macrophages (19, 20). Together, these studies highlight the need for additional studies using state-of-the-art genomic, proteomic, and single cell approaches to clarify the heterogeneity of MDSC and their overlap with other cell types.

Recent studies have provided important information regarding various aspects of metabolic reprogramming in MDSC which contribute to their immunosuppressive effects. In the tumor microenvironment, MDSC display increased accumulation of lipids as well as increased fatty acid oxidation with a switch to glycolysis and a decrease in oxidative phosphorylation (2). Interestingly, under hypoxic conditions, activation of hypoxia-inducible factor 1α (HIF1A) in MDSC was found to induce a switch from oxidative phosphorylation to glycolysis (21, 22)

MDSC were initially characterized functionally based on their ability to suppress T cell proliferation (23). It is now clear that MDSC can suppress other immune cells including B cells and NK cells. PMN-MDSC and M-MDSC mediate their suppressive effects *via* common and unique mechanisms (2). The common mechanisms of suppression include increased expression of signal transducer and activator of transcription 3 (STAT3), induction of ER stress, and expression of arginase-1 (arg-1) and S100A8/A9, which plays a role in cell maturation and trafficking. PMN-MDSC mediate immune suppression *via* production of reactive oxygen species (ROS), peroxynitrite, and arg-1, while M-MDSC produce nitric oxide (NO) and immunoregulatory cytokines including IL-10 and TGF-β. M-MDSC also mediate immunosuppression by up-regulating the expression of immunoregulatory molecules such as PDL1.

### MDSC and Infection

In addition to expansion of MDSC in tumor-bearing mice and cancer patients and their ability to suppress anti-tumor immunity, MDSC expand and suppress immune responses during infections with bacteria and viruses (24). On the other hand, relatively little is known about the role of MDSC in infections with parasites especially helminths (25). Understanding the role of MDSC in infections with bacteria and viruses thus provides insight for investigating the role of MDSC in helminth infections. For this reason, we briefly review what is known about MDSC in these infections.

It is important to point out that the role of MDSC in an infection depends on the classification and virulence of the pathogen, whether the infection is acute or chronic, and the pathology associated with the infection (24). Depending on these variables, different subsets of MDSC may be involved and the mechanism(s) of suppression may differ. MDSC have generally been characterized in infection based on immunophenotyping as well as using functional assays. As will be described in the following sections, the *in vivo* role of MDSC identified by *ex vivo* approaches has been validated by adoptive transfer of purified MDSC from infected animals to naïve hosts, by depletion of MDSC using monoclonal antibodies or drugs targeting these cells, or by inhibitors of relevant immunosuppressive mechanisms. Furthermore, the role of MDSC in infection may be detrimental to successful control of the pathogen by suppressing host immune responses (24). Alternatively, MDSC may benefit the host by controlling inflammation and immunopathology. Ultimately, however, the role of MDSC in a given infection is highly dependent on the identity of the pathogen.

### MDSC and Bacteria

As mentioned above, the role of MDSC in infection is highly determined by the identity of the pathogen and the nature of the disease. Experimental studies in mice and humans indicate that there is marked expansion of CD11b^+^Gr1^+^ cells resembling MDSC in various acute bacterial infections, including *Staphylococcus aureus* (26) and *Klebsiella pneumoniae* (27). Interestingly, MDSC were found to have opposing effects in infections with these pathogens. MDSC exacerbated *S. aureus* infection in mice and human skin by suppressing T cell proliferation. On the other hand, MDSC contribute to resolution of lung inflammation and survival in *K. pneumoniae*-infected mice by efferocytosing apoptotic neutrophils, a response that is partially IL-10-dependent.

Chronic *Helicobacter pylori* infection results in neoplastic transformation of gastric mucosal cells in some patients, but the factors responsible are unclear. In a mouse model of *Helicobacter* infection, Ding et al. identified myeloid-derived cells, which accumulate in the stomach of *H. pylori*-infected mice, that express markers characteristic of PMN-MDSC and suppress T cell proliferation (28). Analyses of MDSC markers in tissue biopsies from patients with gastric cancer demonstrated that cells corresponding to PMN-MDSC are present in the gastric mucosa of *H. pylori*-infected individuals with cancer while MDSC are absent in those without *H. pylori* infection. Based on these findings, the authors concluded that accumulation of PMN-MDSC in the stomach of *H. pylori*-infected patients may be a biomarker for progression to malignancy.

Acute and chronic *Mycobacterium tuberculosis* infections in mice result in the accumulation and expansion of a heterogenous population of MDSC, which express Ly6G or Ly6C indicative of PMN-MDSC or M-MDSC, respectively, in the lung as well as other tissues (29, 30). Interestingly, PMN-MDSC appear to predominate in acute settings while M-MDSC expand in chronic *M. tuberculosis* infection. In both infection settings, the MDSC sub-population that accumulates suppresses CD4^+^ T cell proliferation and IFN-γ production, critical components of protective immunity to *M. tuberculosis*. In humans with active *M. tuberculosis* infection, MDSC increase in the blood and lung and consist of a heterogenous population with a morphology resembling either PMN-MDSC or M-MDSC at various stages of development (31). Furthermore, successful treatment of patients with active *M. tuberculosis* infection is associated with decreased frequencies of MDSC. Similar to observations in *M. tuberculosis*-infected mice, MDSC from infected patients suppress CD4^+^ as well as CD8^+^ T cell proliferation and cytokine production including IFN-γ production.

### MDSC and Viruses

The role of MDSC in viral infections has also been investigated. Infection with Japanese encephalitis virus (JEV), which causes viral encephalitis, induces increases in a population of CD11b^+^Gr1^+^ cells in infected mice (32). These cells suppress CD4^+^ T cell proliferation *in vitro* suggesting that MDSC may contribute to disease in this model. Depletion of CD11b^+^Gr1^+^ cells in JEV-infected mice by administration of retinoic acid, which specifically depletes MDSC, resulted in significant increases in survival of treated mice compared to untreated controls.

Expansion of MDSC has been investigated in mice infected with strains of lymphocytic choriomenigitis virus (LCMV) that cause either an acute or resolving infection (Armstrong strain) or a chronic infection (strain C13) (33). Monocytic cells, including DC, were found to expand to a comparable degree in the spleen during the first week of infection with either the Armstrong or C13 strains. However, high numbers of cells with morphological, phenotypic, and functional characteristics of M-MDSC were sustained in mice infected with the C13 strain (33). Sorted M-MDSC from C13-infected mice were found to suppress CD8^+^ T cell proliferation and function *in vitro via* an IFN-γ-dependent mechanism with up-regulated expression of *iNOS* and increased production of NO. A critical finding in this paper is that antibody depletion of cells expressing Gr1 results in enhanced LCMV-specific CD8^+^ T cell responses in mice with chronic LCMV. Transcriptomic analysis of M-MDSC from C13-infected mice revealed important increases in expression of genes associated with proinflammatory responses as well as genes involved in oxidative stress. Together, these data provide strong evidence that MDSC, especially M-MDSC, contribute to the establishment of chronic viral infections by suppressing T cell-mediated responses critical for immune control of the pathogen.

MDSC have also been characterized in chronic infections with retroviruses. Studies in mice infected with LP-BM5, a retrovirus which causes acquired immunodeficiency in mice, demonstrated infection-induced M-MDSC suppress CD4^+^ T cell and B cell responses (34). Moreover, the severity of disease was found to correlate with the *in vitro* inhibitory activity of LP-BM5-induced MDSC. Expansion of MDSC subsets has also been observed in patients with HIV infection and in simian immunodeficient virus (SIV) infection. In general, these studies indicate that the MDSC subset responsible for suppression of virus-specific immune responses to HIV or SIV varies depending on the phase of the infection. Most studies indicate that PMN-MDSC predominate during these infections. PMN-MDSC were found to increase in patients with primary HIV as well as in patients with chronic HIV (35). Furthermore, these cells inhibited HIV-specific CD8^+^ T cell responses by various mechanisms including expression of PDL1. Remarkably, PMN-MDSC from HIV patients inhibit the proliferation of CD8^+^ T cells from HIV controllers, that is, HIV^+^ individuals who can control viral replication in the absence of anti-retroviral therapy, and from healthy volunteers. In addition, regulatory T cells were found to expand in HIV controllers suggesting immunosuppressive networks involving MDSC and regulatory T cells may converge to influence the final outcome of an infection. Despite effective anti-retroviral therapy, a dramatic increase in the frequency of M-MDSC has also been detected in HIV patients that correlates with various disease markers including viral load (36). These M-MDSC suppress antigen-specific and non-specific responses of CD4^+^ T cells as well as CD8^+^ T cells in a cell-cell contact dependent mechanism that involves arg-1. Interestingly, M-MDSC from HIV patients were found to harbor low copy numbers of HIV DNA, suggesting HIV can infect MDSC (36). This observation was confirmed by *in vitro* studies demonstrating that HIV can infect and replicate in M-MDSC from healthy donors. SIV infection in macaques has also been reported to induce expansion of M-MDSC which suppress CD8^+^ T cell responses (37). Furthermore, anti-retroviral therapy was demonstrated to induce contraction of MDSC in infected macaques.

MDSC frequencies have also been observed to increase in other chronic viral infections, including infections with hepatitis C virus (HCV) and hepatitis B virus (HBV). Studies in humans as well as mice provide evidence of expansion of M-MDSC and PMN-MDSC (35, 38–41). However, the findings indicate the sub-population of MDSC that predominates varies depending on the host and whether the infection is due to HCV or HBV. Overall, the findings reported in studies on HCV and HBV are conflicting and difficult to reconcile as opposed to findings in other chronic viral infections especially chronic LCMV, which provide clear evidence implicating high frequencies of MDSC in chronicity. Additional studies are thus needed to resolve the inconsistency of findings for HCV versus HBV infections.

### MDSC and Helminths

In addition to the ability of helminths to induce the immunoregulatory pathways described above, these parasites appear to be highly adept at inducing the expansion of MDSC (**Table 1**). Prior to phenotypic and morphological characterization of MDSC, it was observed that helminth infections induce suppressive myeloid cell populations with features resembling MDSC. Interestingly, increases in MDSC frequency and number are evident in tissues colonized by the parasite as well as systemically during infection. These findings raise the intriguing possibility that MDSC expand during helminth infection and may contribute to the immunosuppression and chronicity characteristic of infections with parasitic worms.

**Table 1:** Human and zoonotic helminth species inducing differentiation and expansion of MDSC^1^.

Species	Tissue site	Cellular Response	Suppression Observed	References
Human trematode (flukes) *Schistosoma mansoni*	Bone marrow Liver, spleen Spleen	↑Myelopoiesis during early infection^2^ ↑Myeloid-derived cells during chronic infections ↑ CD11b^+^Gr1^+^MHC-II^-^ CD16^+^F4/80^dull^ cells	ND^3^ ND ↓Antigen and allo-specific CD8^+^ T cell proliferation^2^	42–44 44, 45 46
*Schistosoma japonicum*	Bone marrow, spleen, mesenteric lymph node	↑ CD11b^+^Ly6G^+^Ly6C^-/low^ IL-10^+^ cells	↓ CD4^+^ and CD8^+^ T cell proliferation via ROS production	47
Zoonotic cestodes (tapeworms) *Taenia crassiceps*	Peritoneal cavity	↑ CD11b^+^Gr1^+^ cells during chronic phase	↓ Polyclonal T cell proliferation via ROS production	48, 49
*Mesocitoides vogae*	Peritoneal cavity, spleen	↑ CD11b^+^Gr1^+^ F4/80^low^MHC-II^low^ Ly6G^+^ cells during chronic infection	↓ T cell proliferation	50
Mouse nematodes (round worms) *Heligmosomoide polygyrus bakeri*	Mesenteric lymph node, spleen Lamina propria Bone marrow, blood, peritoneal cavity	↑ CD11b^+^Gr1^hi^ F4/80^-^Ly6G^+^ Ly6C^+^ cells ↑ CD11b^+^Gr1^hi^ F4/80^-^Ly6G^+^ cells ↑ CD11b^+^Gr1^+^ Ly6C^dim^ cells	↓ OVA-specific CD4^+^ T cell proliferation via NO production; ↓Spleen cell IL-4 production in response to parasite antigens ND ↓Polyclonal CD4^+^ T cell proliferation via arginase-1	51 51 52 53, 54
*Nippostrongylus brasiliensis*	Spleen	↑ CD11b^+^Gr1^+^ Ly6G^+^ cells ↑ CD11b^+^Gr1^+^ Ly6C^+^ cells	ND	

^1^ Data presented based on studies in inbred mice.

^2^ ↑, indicates increased response; ↓, indicates decreased response.

^3^ND, not determined.

### MDSC and Trematodes (*Schistosoma* species)

Expansion of MDSC and their contribution to suppression of immune responses have been investigated extensively in mice infected with schistosomes, the causative agent of human schistosomiasis. The major pathology associated with schistosome infection in humans and laboratory mice is hepatomegaly with granuloma formation and fibrosis in the infected liver and splenomegaly. Several early studies indicate myelopoiesis is increased in the bone marrow during acute *Schistosoma mansoni* infection (42, 55). During chronic *S. mansoni*, bone marrow myelopoiesis was found to return to normal levels while myelopoiesis increased in the liver as well as the spleen as the infection progressed to the chronic stage (48). A study tracking GFP^+^ cells in bone marrow chimeras infected with *S. mansoni* demonstrated the majority of CD45^+^ cells migrating to the liver of infected recipient mice localized in granulomas and are myeloid cells that express CD11b but are Gr1^-^ suggesting the cells are macrophages (56). However, no further phenotypic or functional characterization of these cells was undertaken. Based on extensive immunophenotyping of spleen cells from mice chronically infected with *S. mansoni*, Marshall and colleagues found that CD11b^+^Gr1^+^MHC-II^-^CD16^+^F4/80^dull^ cells expand dramatically in infected spleens (43). Furthermore, these cells, phenotypically similar to M-MDSC, were demonstrated to suppress viral peptide-specific and allo-specific CD8^+^ T cell proliferation *in vitro*. Altogether, these studies indicate that MDSC may increase during infection with schistosomes.

Determination of the frequency and number of cells expressing markers consistent with MDSC in the bone marrow, spleen, and mesenteric lymph node (MLN) of *S. japonicum*-infected mice provide compelling evidence that MDSC increase during helminth infection (47). This study used Ly6C and Ly6G as markers to further delineate the MDSC sub-populations that increased in infected mice. Using a panel of well-defined markers of MDSC to immunophenotype cellular responses, the investigators observed that the majority of MDSC in tissues of mice infected with *S. japonicum* are CD11b^+^Ly6G^+^Ly6C^-/low^. This is consistent with currently accepted phenotypic identification of PMN-MDSC. M-MDSC expressing Ly6C also increased compared to normal, uninfected mice. However, PMN-MDSC increased significantly while increases in M-MDSC were not significant in infected compared to uninfected mice. Furthermore, PMN-MDSC from infected mice suppress ConA-induced proliferation of CD4^+^ and CD8^+^ T cells *in vitro*. Intracellular cytokine staining showed CD11b^+^Ly6G^+^Ly6C^-/low^ cells from infected mice express cytokines, including IL-10, known to be secreted by MDSC during bacterial infections. In addition, suppression of T cell responses by PMN-MDSC was found to be associated with dramatic induction of the NADPH oxidase sub-units gp91^phox^ and p47^phox^ and occurred *via* production of ROS.

This study also reported that soluble egg antigen and somatic antigen from adult worms of *S. japonicum* induce MDSC *in vitro* in bone marrow cultures (47). Furthermore, increased expression of S100A8/9, associated with the generation of MDSC, was dramatically increased in MDSC induced *in vitro* by worm products and in MDSC purified from infected mice. Moreover, the suppressive effects of MDSC from infected mice and MDSC induced *in vitro* by worm products were found to be dependent on signaling *via* the JAK/STAT3 pathway, which is important in maintaining the suppressive function of MDSC (2). A possible mechanism used by *S. mansoni* and possibly *S. japonicum* to recruit MDSC to the liver and other tissues during infection may be immunoregulatory oligosaccharides, specifically lacto-*N*-fucopentaose III and lacto-*N*-neotetraose, expressed on egg antigen (44). Injection of these molecules into the peritoneum of naïve mice was reported to induce rapid recruitment of a population of highly suppressive CD11b^+^Gr1^+^F4/80^+^ cells (45, 46). Thus, products of schistosomal parasites and other helminths, as will be described below, induce the accumulation of MDSC *in vitro* and *in vivo* (**Table 2**).

**Table 2:** Helminth products that induce MDSC differentiation and expansion *in vivo* and *in vitro*.

Species	Products	*in vitro*/*in vivo* sites	References
*Schistosoma japonicum*	Soluble egg antigen, Somatic worm antigen	Bone marrow cultures	47
*Schistosoma mansoni*	Oligosaccharides	Peritoneal cavity	57–59
*Taenia crassiceps*	Glycans	Peritoneal cavity	49, 60
*Mesocitoides vogae*	ES products	Peritoneal cavity	50
*Brugia malayi*	ES products	Peritoneal cavity	56
*Nippostrongylus brasiliensis*	ES products	Peritoneal cavity	56

### MDSC and Cestodes (Tapeworms)

The tapeworm, *Taenia crassiceps*, is a cestode parasite found in its adult form in the small intestine of canids whereas the main larval stage or metacestode is found in the muscle as well as the pleural and peritoneal cavities of rodents including mice (57). Implantation of *T. crassiceps* larvae in the peritoneum of mice induces the accumulation of elevated numbers of CD11b^+^Gr1^+^ cells as the infection progresses (58). These cells were found to suppress the proliferation of naïve T cells stimulated with anti-CD3/CD28 antibodies *in vitro*. During early infection, T cell suppression by these cells is NO-dependent. Later, however, CD11b^+^Gr1^+^ cells recovered from the peritoneum of mice implanted with *T. crassiceps* produce high amounts of arg-1 and low levels of NO and suppress T cell function *via* production of ROS, including H_2_O_2_ and superoxide reminiscent of PMN-MDSC. Similar to antigens from *S. japonicum* and oligosaccharides from *S. mansoni*, glycans from *T. crassiceps* induce the accumulation of cells *in vivo* that express MDSC markers and suppress T cell proliferation (59).

Another cestode *Mesocitoides vogae*, previously known as *M. corti*, which colonizes the GI tract of carnivores including dogs and, in rare cases, causes human tapeworm infections, has also been reported to induce MDSC (49). Larval stages of *M. vogae* can also multiply in the liver and peritoneal cavity of intermediate hosts including rodents. Oral infection of mice with second stage larvae or metacestodes modulates cell populations and immune responses in the peritoneal cavity and provides a useful model to study host-parasite interactions. Using this approach, Mačak-Kubašková and colleagues observed CD11b^+^Gr1^+^F4/80^hi^MHC-II^hi^ cells increase in the peritoneal cavity during the first 10 days after *M. vogae* infection together with increases in the Th1 cytokine IFN-γ in peritoneal lavage fluid (49). These cells produce high levels of NO and inflammatory cytokines in response to LPS *in vitro* and thus resemble classically activated macrophages. Later in infection, CD11b^+^Gr1^+^F4/80^low^MHC II^low^ cells increase and predominate in the peritoneal cavity and expand in the spleen as well. Cells in this population were found to express Ly6C or Ly6G indicating expansion and accumulation of both M-MDSC and PMN-MDSC in infected mice. Purified Ly6C^+^ and Ly6G^+^ cells were found to suppress polyclonal T cell proliferation *in vitro*. Ly6G^+^ cells, which resembled PMN-MDSC morphologically and phenotypically, had higher suppressive activity than Ly6C^+^ cells. Moreover, these cells produce IL-10 and express *Arg-1*. In addition, STAT3 expression is up-regulated in Ly6C^+^ cells. Similar to *T. crassiceps*, intraperitoneal injection of *M. vogae*-derived ES induces accumulation of cells consistent with the phenotype of MDSC and these cells express intracellular IL-10.

### MDSC and Nematodes

In addition to accumulation of MDSC in the tissues of hosts infected with schistosomes and tapeworms, nematodes that infect various tissues including the intestine also induce the expansion of these cells. Infection with *Brugia malayi*, a causative agent of lymphatic filariasis in humans, results in edema of lymphatic tissue, leading to elephantiasis, a severe form of chronic *B. malayi* infection (8). Intraperitoneal implantation of adult stages of *B. malayi* in mice results in the appearance of a suppressive population of myeloid cells that are adherent and inhibit antigen-specific CD4^+^ T cell proliferation (52). This finding may provide a mechanistic explanation for the hyporesponsiveness of T cells to polyclonal and antigen-specific stimuli characteristic of individuals infected with *B. malayi* (60). In line with observations described above that helminth products can induce the accumulation of suppressive myeloid cells *in vivo*, daily intraperitoneal injection of ES products derived from *B. malayi* as well as from *Nippostrongylus brasiliensis* induce suppressive cells that express myeloid markers and inhibit antigen-specific CD4^+^ T cell proliferation (50). As will be discussed below, infection with *N. brasiliensis*, a gastrointestinal nematode of rodents that migrates to the lung prior to colonization of the intestine, induces MDSC sub-populations expressing PMN-MDSC and M-MDSC markers (61). While these findings are intriguing and highlight the importance of helminth-derived products in inducing MDSC, additional studies are required to determine the identity of helminth-derived ES products that induce MDSC and their mechanism(s) of action.

Infection with the GI nematode *H. polygyrus bakeri* has also been demonstrated to induce the expansion of MDSC. *H. polygyrus bakeri*, a natural pathogen of mice, is one of the most widely used laboratory models to study immune responses to intestinal nematodes that infect humans and livestock. *H. polygyrus bakeri* is closely related to the human hookworm species *N. americanus* and *A. duodenale*. Similar to other helminths, these nematodes establish chronic infections in their host despite the ability to induce a highly polarized Th2 immune response (62–64). Adult worms of GI nematodes are eliminated by administration of an anthelmintic drug. This treatment results in a highly effective Th2 immune response to a challenge infection, that is, reinfection, with *H. polygyrus bakeri*, a scenario unlike the lack of specific immunity to reinfection apparent in human helminth infections.

The Th2 cytokine IL-4 produced primarily by CD4^+^GATA4^+^ T cells is essential for expulsion of adult worms during primary infection (6). It should be pointed out that adult worm burdens are significantly higher after primary *H. polygyrus bakeri* infection versus drug-cure and challenge, that is, reinfection (51). This suggests that Th2 immunity may be suppressed during primary infection. Here, we highlight major findings from our recent work investigating this possibility in C57BL/6 mice after primary and challenge infections with *H. polygyrus bakeri*. We observed that CD4^+^GATA3^+^ T cells increased significantly in the MLN after both primary and challenge infections compared to uninfected mice, but their numbers were significantly lower after primary infection compared to challenge infection. Notably, we observed dramatic increases in CD11b^hi^Gr1^hi^F4/80^-^ cells in the MLN and spleen after primary *H. polygyrus bakeri* infection while there were no significant increases in this cell population after challenge infection (51, 65). This indicates that MDSC expand at the site of infection as well as systemically after primary infection but not after challenge infection. Surprisingly, we found that the predominant sub-population of MDSC in the tissues of mice during primary *H. polygyrus bakeri* infection express both Ly6G and Ly6C, that is, the CD11b^+^Gr1^+^ cells in infected mice are Ly6G^+^Ly6C^+^. There were no significant increases in the Ly6G^-^LyC^+^ population in the MLN or spleen while increases in Ly6G^+^LyC^-^ cells were relatively small although the frequency was significantly higher compared to naïve mice. CD11b^hi^Gr1^hi^F4/80^-^ cells also increased in the lamina propria (LP) during primary infection indicating MDSC accumulate in the infected intestine. Moreover, cells with immature morphology resembling MDSC were apparent in the LP and spleen of mice during primary infection. After primary *H. polygyrus bakeri* infection, MDSC increased within days of infection in the MLN and spleen, while significant increases in alternatively activated macrophages, which are required for expulsion of adult worms, were delayed (7). In addition, the number of alternatively activated macrophages in the LP of infected mice after primary infection did not increase significantly compared to naïve mice. Together, these data demonstrate that MDSC increase in intestinal and systemic lymphoid tissue during primary *H. polygyrus bakeri* infection. Furthermore, accumulation of MDSC in the infected intestine early after infection may negatively regulate macrophage polarization to alternatively activated macrophages.

To determine if *H. polygyrus bakeri*-induced MDSC suppress antigen-specific effector CD4^+^ T cell responses, we purified CD11b^+^Gr1^+^ cells and co-cultured them with spleen cells from OT-II mice. CD11b^+^Gr1^+^ cells from mice with primary *H. polygyrus bakeri* infection potently suppress CD4^+^ T cell proliferation *in vitro* in response to the MHC class II peptide OVA_323-339_ *via* a NO-dependent mechanism (51). These cells, which phenotypically and morphologically resemble MDSC, also significantly suppressed IL-4 secretion by spleen cells from infected mice in response to parasite antigen in a dose dependent manner. Adoptive transfer of purified CD11b^+^Gr1^+^ cells from infected mice to naïve recipients demonstrated higher adult worms burdens and increased egg production in recipient mice after infection with *H. polygyrus bakeri*. Thus, MDSC from mice with primary *H. polygyrus bakeri* infection suppress Th2 responses in *in vitro* and vivo.

A study performed in BALB/c mice confirmed that MDSC expand during primary *H. polygyrus bakeri* infection (65). This paper demonstrated that CD11b^+^Gr1^+^ cells increase in the bone marrow, blood, and peritoneal cavity early after infection. Larval stages were found to induce MDSC in the intestinal wall of infected mice. Moreover, Gr1^+^ cells purified from the MLN and spleen of infected BALB/c mice suppressed CD4^+^ T cell proliferation. Importantly, depletion of Gr1^+^ cells in the cultures reversed suppression of T cell responses. It was also shown that *Arg-1* and *iNos* expression are up-regulated in MDSC recovered from the spleen and peritoneal cavity of infected mice. Determination of the role of arginase in MDSC-mediated immunosuppression during *H. polygyrus bakeri* infection was investigated *in vitro* by the addition of L-arginine or inhibition of arg-1 in the T cell suppression assays and *in vivo* by administration of L-arginine or arg-1 inhibitor. Modulation of arginine or its metabolism, critical for NO production, significantly reversed CD4^+^ T cell suppression *in vitro* and significantly reduced the adult worm burden *in vivo*. Together, these studies in *H. polygyrus bakeri*-infected C57BL/6 and BALB/c mice provide robust evidence that MDSC are induced to expand during GI nematode infection and suppress Th2 responses essential for expulsion of adult worms.

As mentioned above, *N. brasiliensis* induces increases in MDSC in infected mice. Furthermore, these cells were found to express Ly6G or Ly6C, with Ly6G^+^ cells being the prominent sub-population (61). In contrast to primary infection with *H. polygyrus bakeri*, which is chronic unless parasites are removed by treatment with an anthelmintic drug, infection with *N. brasiliensis* is acute in immunocompetent mice and adult worms are expelled (7). The authors proposed that increases in MDSC may result in enhanced expulsion of adult worms in *N. brasiliensis*-infected mice (53, 61). Indeed, expansion of MDSC in *N. brasiliensis*-infected mice was associated with adult worm expulsion and increases in Th2 cytokines IL-4, IL-5, and IL-13 in sera. The authors also observed increased serum levels of IL-17, previously reported to be produced by MDSC in tumor-bearing mice (54, 61). Importantly, treatment of *N. braseliensis*-infected mice with the chemotherapeutic drug gemcitabine to deplete MDSC resulted in higher adult worm burdens in the intestine and increased egg production, a measure of adult worm fecundity (53, 61). This finding may provide a rationale for treating similar helminth infections in humans with MDSC-depleting drugs such as gemcitabine, but additional studies are required. In addition, adoptive transfer of purified Ly6G^+^ cells from infected mice to naïve recipients resulted in decreased egg production similar to adoptive transfer of Gr1^+^ cells. In a separate study from the same investigators, gemcitabine depletion of MDSC in mice infected with the nematode *Trichinella spiralis*, which is responsible for trichinosis in humans, resulted in significant increases in adult worms in the intestine as well as subsequent development of larvae in the muscle of infected mice. These findings support the notion that MDSC may be beneficial during infections with some helminths, including *N. braseliensis* and *T. spiralis*.

## Conclusion and Future Perspectives

**Figure 1:**
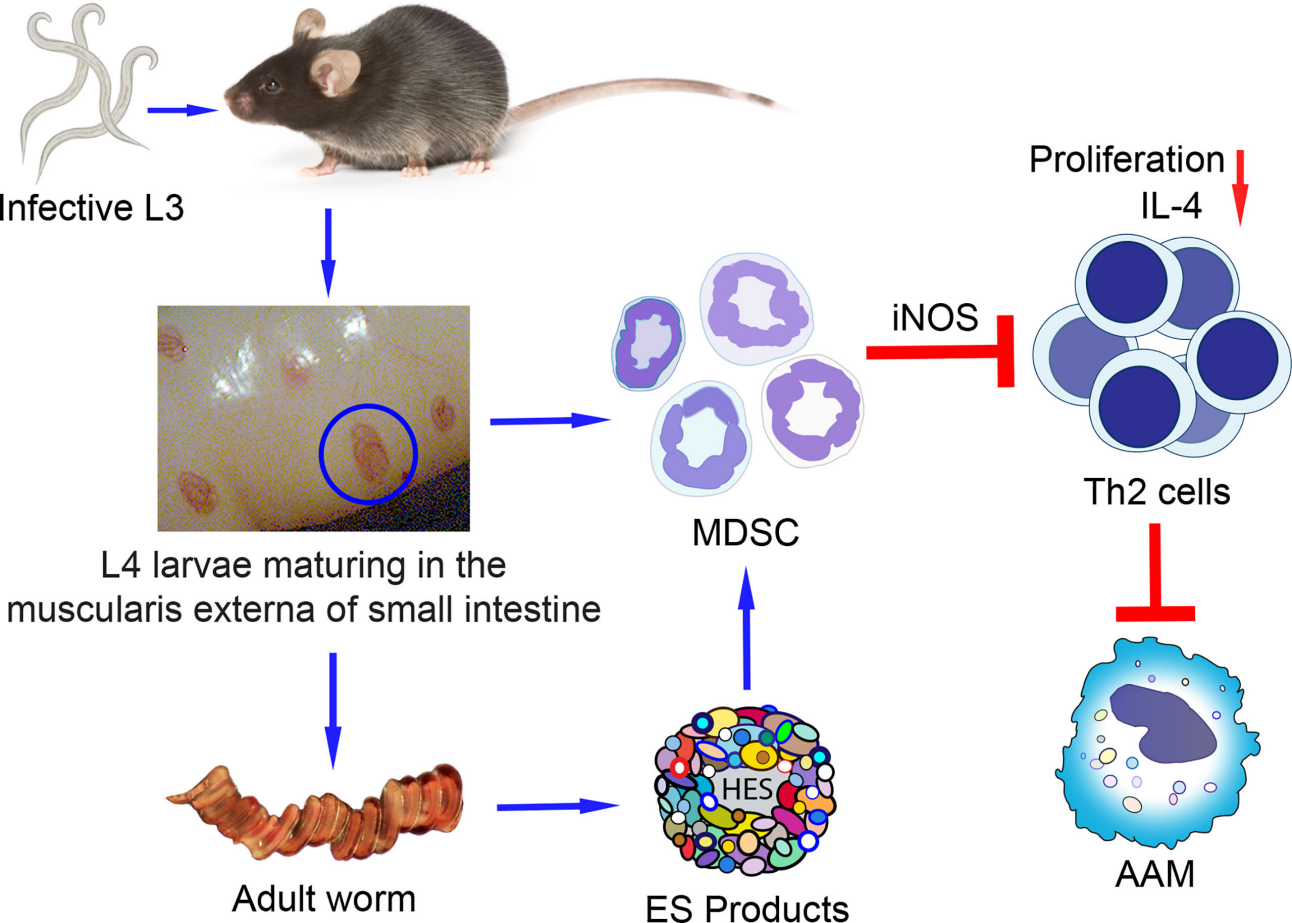
Proposed model of MDSC differentiation and expansion induced by the GI nematode using *H. polygyrus bakeri* as an example. Mice are infected with *H. polygyrus bakeri* by ingestion of infective stage 3 larvae (L3) which develop into stage 4 larvae (L4) in the muscularis externa of the small intestine. L4 eventually mature into male and female adult worms that mate and produce eggs which are shed in the feces and contaminate the soil. Larvae and adult worms induce the differentiation and expansion of MDSC in the infected intestine and systemically in distal tissues. The MDSC suppress the proliferation of CD4^+^ Th2 cells and their IL-4 secretion in response to parasite-specific antigens. Decreased levels of IL-4 result in sub-optimal numbers of alternatively activated macrophages (AAM) important in expelling adult worms and in immunity to reinfection. ES (HES from *H. polygyrus bakeri*) produced by adult worms or larvae may also induce MDSC.

Similar to infections with other pathogens, accumulating evidence indicates that myeloid-derived cells with morphologic, phenotypic, and suppressive functions characteristic of MDSC expand during infections with helminth parasites (**Figure 1**). Given the unique and complex life cycles of parasitic worms as well as the diseases they cause, it is not surprising that expansion of MDSC is evident at the site of infection and in systemic lymphoid tissue. Likewise, it is not surprising that the sub-population of MDSC which predominates at various times after infection with these pathogens varies and that the predominate sub-population of MDSC is highly dependent on the infecting helminth species. As highlighted in this review, the mechanism(s) of suppression mediated by MDSC also appears to vary among the helminths studied to date. Interestingly, it has been found that MDSC are beneficial during some helminth infections.

Together, the findings reported to date regarding MDSC and helminths raise numerous questions that require further investigation. These questions include the identification of growth factors responsible for the expansion of MDSC in the context of a given helminth infection, the transcription factors involved, and the transcriptional cues which dictate differences in the potency of immunosuppression in Ly6G^+^ versus Ly6C^+^ MDSC sub-populations reported in various helminth infections. Other intriguing and outstanding questions regarding MDSC and helminth infection revolve around the ability of helminth-derived products including ES products to induce the expansion and accumulation of MDSC at the site of infection as well as in distal lymphoid tissue. Areas that require further investigation are whether there is a universal role for helminth-derived products in MDSC expansion across the diversity of parasitic worms or if these products are more important drivers of MDSC in some types of helminth infections than others. Studies are also required to identify the molecule(s) in ES and other helminth-derived products that induce MDSC. It is also very intriguing why the predominant sub-population of MDSC changes with time during helminth infections and whether this represents MDSC differentiation during these often long lasting and chronic infections.

Finally, studies are required to investigate MDSC in human populations where helminth infections are endemic and to explore the effects of various drugs and inhibitors reported to delete MDSC or inhibit their immunosuppressive effects as therapeutic approaches in helminth infections. The combination of MDSC-targeted agents and anthelminthic drugs may be effective in eliminating adult worms and inducing immunity to reinfection. Thus, a clearer understanding of MDSC in helminth infection holds promise for controlling these important and devastating NTDs in the developing world.

## Author Contributions

MS wrote the manuscript and revised the final manuscript. MT contributed to the writing, reviewed the manuscript, and prepared the figure. RM contributed to the research and reviewed the final manuscript. All authors contributed to the article and approved the submitted version.

## Funding

The authors gratefully acknowledge funding from the Canadian Institutes of Health Research (MOP130369 to MS), National Science and Engineering Research Council of Canada (1327730 to MS), and Fonds de recherche du Québec: Nature et Technologies (253419 to MS).

## Conflict of Interest

The authors declare that the research was conducted in the absence of any commercial or financial relationships that could be construed as a potential conflict of interest.

## Publisher’s Note

All claims expressed in this article are solely those of the authors and do not necessarily represent those of their affiliated organizations, or those of the publisher, the editors and the reviewers. Any product that may be evaluated in this article, or claim that may be made by its manufacturer, is not guaranteed or endorsed by the publisher.

## Footnotes

1 https://www.cdc.gov/globalhealth/ntd/diseases/index.html.
